# Cold Food in Winter

**Published:** 1891-04

**Authors:** 


### COLD FOOD IN WINTER.

A writer in *Farm and Home* (English), thus expresses himself upon this subject. The writer was asked out to "supper" two or three weeks ago. He wondered a little why it was supper and not dinner. When supper appeared the roast beef was cold, and so was the beetroot; the claret also was cold; there were cold blancmanges and cold mince pies; and after that cold biscuits and cheese. The thermometer outside marked about 9 deg. of frost. The host was a man of high scientific attainments, and his wife was a clever woman and an excellent housekeeper. That cold collation evoked, at the beginning, a suppressed groan, which later, when the troubled spirit had reached the safe shelter of its own bedroom, developed into an unsuppressed malediction. The cold supper was heavy as lead, and almost as indigestible. The event was forgotten, however, until about a fortnight later, when a friend of the writer's was invited to the house of a medical man; a man whose wife had been a hospital nurse. This physiological husband and wife also gave a supper instead of a dinner, and it, too, was cold "from the eggs to the apples." These two events happening within a fortnight of each other, and in the coldest winter that has been experienced for half a century, seemed to indicate that is was a not uncommon thing even for educated people to take meals in the winter which are cold from beginning to end. What to say on such a subject as this that shall be sufficiently expressive is not very apparent. A physiologist, who is not merely a book physiologist, holds up his hands in amazement! No doubt there are happy souls, or, rather, souls that have happy stomachs, that can digest cold beef, cold beetroot, cold blancmange, cold claret, cold cheese, cold biscuits, and cold butter, all piled on the top of one another, even in an arctic climate, and when the ice is a foot thick on the upper reaches of the Thames. But surely these miraculous stomachs are rare! The ordinary man, and particularly the town man, should no more sit down to such a supper, in a winter like the present, than he should to a supper of Wenham ice cut into little blocks and served with parsley.

